# Lumbar spine MRI findings in asymptomatic elite male academy footballers: a case series

**DOI:** 10.1186/s13102-022-00576-1

**Published:** 2022-10-24

**Authors:** Sean Carmody, Gajan Rajeswaran, Adam Mitchell, Katrine Okholm Kryger, Imtiaz Ahmad, Munraj Gill, Alison Rushton

**Affiliations:** 1 School of Sport, Exercise and Rehabilitation Sciences, University of Birmingham, Birmingham, UK; 2 Medical Department, Queens Park Rangers Football and Athletic Club, London, UK; 3 OneWelbeck, London, UK; 4 Faculty of Sport, Allied Health and Performance Science, St Mary’s University, Twickenham, London, UK; 5 Croydon University Hospital, Croydon, UK; 6 Centre of Precision Rehabilitation for Spinal Pain, School of Sport, Exercise and Rehabilitation Sciences, University of Birmingham, Birmingham, UK; 7 Amsterdam UMC location, University of Amsterdam, Department of Orthopedic Surgery and Sports Medicine, Meibergdreef 9, Amsterdam, The Netherlands

**Keywords:** Imaging, Soccer, Football, Youth, Injury

## Abstract

**Background:**

Understanding common MRI findings may allow clinicians to appreciate the sport-specific effects on the lumbar spine, and to discern clinically significant pathology. Prevalence data regarding radiological abnormalities seen during the surveillance of asymptomatic elite footballers is, therefore, important to help understand injury mechanisms and to prevent associated injuries. The purpose of this study was to evaluate the magnetic resonance imaging (MRI) findings in the lumbar spines of asymptomatic elite male adolescent footballers.

**Methods:**

A prospective case-series study was carried out. MRI was performed using a 3T Siemens Prisma scanner including a 3D VIBE sequence in 18 asymptomatic male elite adolescent footballers recruited from a professional academy in England (mean age 17.8, range 16.9–18.6 years). The images were independently reported by two consultant musculoskeletal radiologists to achieve consensus opinion. Standardised classification criteria were used to assess and report abnormalities descriptively.

**Results:**

Fifteen players (15/18, 83%) showed ≥1 abnormalities, included facet degeneration, synovial cysts, disc degeneration, disc herniation, and pars injury. One player (1/18, 6%) had mild (Grade 1) facet joint arthropathy at L4/L5 and 3/18 (16.7%) showing evidence of bilateral facet joint effusions. Three synovial cysts were identified in 2/18 players (11%), 4/18 players (22%) presented asymptomatic pars injuries, with 4 showing a grade 2 subtotal stress fracture and 1 player a grade 4 chronic stress fracture seen on 3D VIBE sequencing. Disc degeneration at one or more levels was demonstrated in 7/18 players (38%). Disc herniation was present in 5/18 players (27%).

**Conclusion:**

A range of unsuspected findings on MRI of the lumbar spine are common in elite adolescent footballers.

## Key Points

- Asymptomatic lumbar spine pathology is a common (83.3%) MRI finding among elite adolescent footballers.
- Lumbar spine pathology does not appear to be as prevalent among footballers as it is among athletes from other sports which place greater demands on the spine (e.g. cricket, tennis).
- Clinicians should interpret positive imaging findings in this population with caution, as they may not correlate to clinical presentation.
- MRI 3D VIBE may be useful to diagnose and assess healing of pars injuries in elite footballers, and could negate the need to expose players to ionising radiation (e.g. CT).

## Background

Academy footballers are at risk of overuse lumbar spine injuries due to the repetitive stresses of flexion, extension, rotation, and lateral flexion that occur during sport-specific movements such as heading, jumping, and tackling. The high training loads with multidirectional stress on the lumbar spine during times of growth are likely to elevate injury risk [1]. There is, however, limited epidemiological data available for more severe injuries such as stress fractures of the pars interarticularis in elite male academy players. It appears that these injuries may carry a significant burden for male Academy football players [2]. It is also known that these issues continue to be troublesome for senior players with low back pain having shown to account for 2% of all injuries in elite men’s football [3]. It must be noted that structural changes to the spine (e.g. degenerative issues) are not necessarily reported in epidemiological studies and therefore, the incidence and severity are poorly understood.

Abnormal findings are not uncommon when performing magnetic resonance imaging (MRI) in asymptomatic adolescent athletes [4–7]. These findings may relate to intense training loads at a time when the spine is undergoing continuous growth and remodelling, making it more susceptible to injury [8]. Earlier studies examining the lumbar spines of elite adolescent tennis players demonstrated a high incidence of radiological abnormalities in asymptomatic subjects [4–7]. While ankle, knee and hip pathologies are well described in elite male academy footballers, [1, 2]. research describing atraumatic spinal injuries in this demographic is limited – especially with respect to radiographic evidence of spinal pathology.

Insight into common MRI findings allow clinicians to appreciate the sport-specific effects on the spine, and to discern clinically significant pathology. Prevalence data regarding radiological abnormalities seen during the surveillance of asymptomatic elite footballers is, therefore, important to help understand injury mechanisms and to prevent associated injuries [9]. Knowledge about the MRI appearances in the lumbar spines of male Academy footballers can contribute to better decisions about preventing and managing injuries. The purpose of this study was to describe the MRI appearances in the lumbar spines of asymptomatic adolescent male footballers at a professional football club academy in England.

## Methods

A prospective case-series study was performed. In order to maintain validity throughout the process, we sought to adhere to the checklist of 22 items outlined in the Strengthening the Reporting of Observational Studies in Epidemiology (STROBE) statement [10]. The procedures for study design, data collection and analysis were comparable to earlier studies performed in adolescent tennis players [4, 7].

### Participants

Eighteen male participants between the ages of 16 and 18 (mean age 17.8, range 16.9–18.6 years) contracted to a single professional football academy in England were recruited. The recruitment was conveniently undertaken from a single squad within the club. The participants included 2 goalkeepers, 10 defenders, 5 midfielders and 1 attacker. None of these players had a cardiac pacemaker, cochlear implant, aneurysm clips (brain), or other bodily metalwork. Importantly, none of the players had any clinical signs or symptoms suggestive of lumbar spine pathology (e.g. lower back pain, radicular symptoms).

#### Inclusion criteria

Male.

Aged 16–18.

Contracted to a single professional club in England.

#### Exclusion criteria

Any bodily metal work (e.g., cardiac pacemaker, aneurysm clips) which would preclude a safe MRI.

No clinical signs or symptoms of lumbar spine pathology (e.g., lower back pain, radicular symptoms).

### Ethics

A risk assessment was performed prior to proceeding with the study in order to identify possible associated risks, their likelihood, and their potential impact on stakeholders. Ethical approval was obtained from the Ethical approval was obtained from the University of Birmingham School of Sport and Exercise Science ethics committee (MCR040219-1). Participants were issued a Participant Information Sheet and provided informed written consent prior to data collection. Incidental and untoward findings were managed according to The Royal College of Radiologists’ guidelines on the *Management of Incidental Findings During Research Imaging* [11]. For example, if unsuspected findings were deemed of clinical significance, arrangements were made to share this information with the player through discussion with the medical team, and where appropriate, further investigations were arranged.

### Data Collection

Imaging was carried out over two separate days of data collection at (redacted for blinded manuscript), using a 3T Siemens Prisma scanner. Sagittal T1, T2 and FS (fat-saturated) T2 sequences were obtained as well as axial T2 sequences through the lower three levels and a 3D T1 spoiled gradient echo VIBE (Volumetric Interpolated Breathhold Examination) sequence. The 3D T1 VIBE sequence is isotropic and of thin slice section allowing multiplanar reformatting and relatively high-resolution evaluation of the pars interarticularis, similar to computed tomography (CT). It potentially negates the need to expose athletes to ionising radiation as is the case when using CT to exclude or further evaluate pars interarticularis fractures. [12]. In two patients, the pars interarticulares were not completely imaged on the sagittal sequences as the MRI technician had not protocolled the sequence to image sufficiently laterally. Although the pars interarticulares were imaged completely on the remaining sequences (including the 3D T1 spoiled gradient echo VIBE sequence), these two participants were recalled for completion sagittal sequences.

### Data Analysis

Two consultant musculoskeletal radiologists (redacted), both with over a decade of experience, reviewed the images independently with findings agreed by standard consensus. Each MRI scan was assessed according to the following categories; facet joint arthropathy, presence of synovial cysts, pars abnormalities, disc abnormalities, spinal cord abnormalities and any other radiological findings as per previous studies [4, 7].

Abnormalities were reported using validated classification criteria. Facet joint arthropathy was classified using a system developed by *Weishaupt et al.*, with grades classed as normal (grade 0), mild (grade 1), moderate (grade 2), or severe (grade 3) [13]. Synovial cysts were described as rounded, fluid containing lesions related to the facet joint, found in the epidural, foraminal, or paravertebral positions. Pars injuries were assessed using a classification system by *Alyas et al.*.^7^ which evolved from a system originally proposed by *Hollenberg et al.* [14]. They were described as normal (grade 0); chronic stress reaction (grade 1); subtotal stress reaction (grade 2); acute stress fracture (grade 3); or chronic stress fracture (grade 4). Disc degeneration was reported according to classification criteria by *Pfirmann et al.* (grade 1 to grade 5; normal to severe) [15]. Disc herniations were described as circumferential (> 50% of the disc circumference); broad-based herniation (25–50% of the disc circumference), central herniation (< 25% of the disc circumference), extrusion or sequestration [16]. Modic end plate changes were recorded if present as type 1 to 3 [17]. Indentation of the thecal sac was noted as present or absent. Nerve root involvement was noted as none, contact but no deviation, deviation and compression. The presence of intervertebral disc herniations, such as Schmorl’s nodes and limbus vertebrae, were also documented. These classifications enabled comparison of results with existing literature in different sports.

## Results

**Fig. 1:**
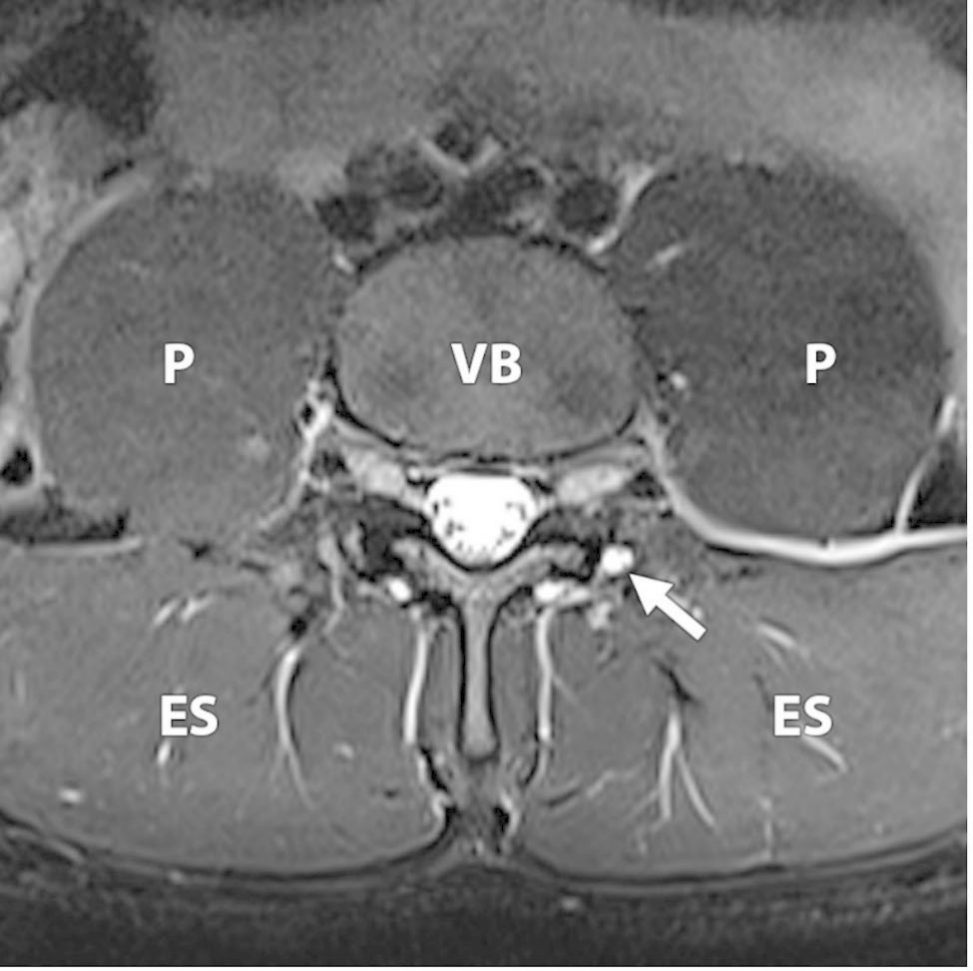
18-year-old male professional football player with no symptoms. Axial fat saturated (FS) T2 sequence showing a rounded high signal lesion with a thin low signal rim consistent with a synovial cyst arising from the posterior left L4/L5 facet joint (white arrow). The vertebral body (VB); psoas muscles (P) and erector spinae muscles (ES) are demonstrated

**Fig. 2:**
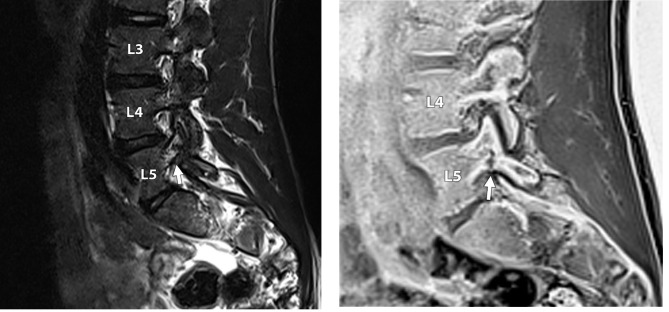
**(a)** 17-year-old male professional football player with no symptoms. **(a)** Sagittal T2 sequence demonstrating high signal in the left L5 pedicle and pars interarticularis indicating acute bone stress (white arrow). There is a suggestion of a low signal fracture line, but this is not definitive. **(b)** 17-year-old male professional football player with no symptoms. **(b)** Sagittal 3D T1 spoiled gradient echo VIBE sequence demonstrating a definitive fracture line in the left L5 pars interarticularis (white arrow) in the region of bone marrow oedema shown in the T2 sequence **(a)**. This extends posterosuperiorly from the inferior cortex of the pars but does not reach the superior cortex in keeping with an incomplete fracture. The fracture margins are indistinct which in combination with the bone oedema seen in **(a)** confirms that this is an acute incomplete pars stress fracture (grade 2)

**Fig. 3:**
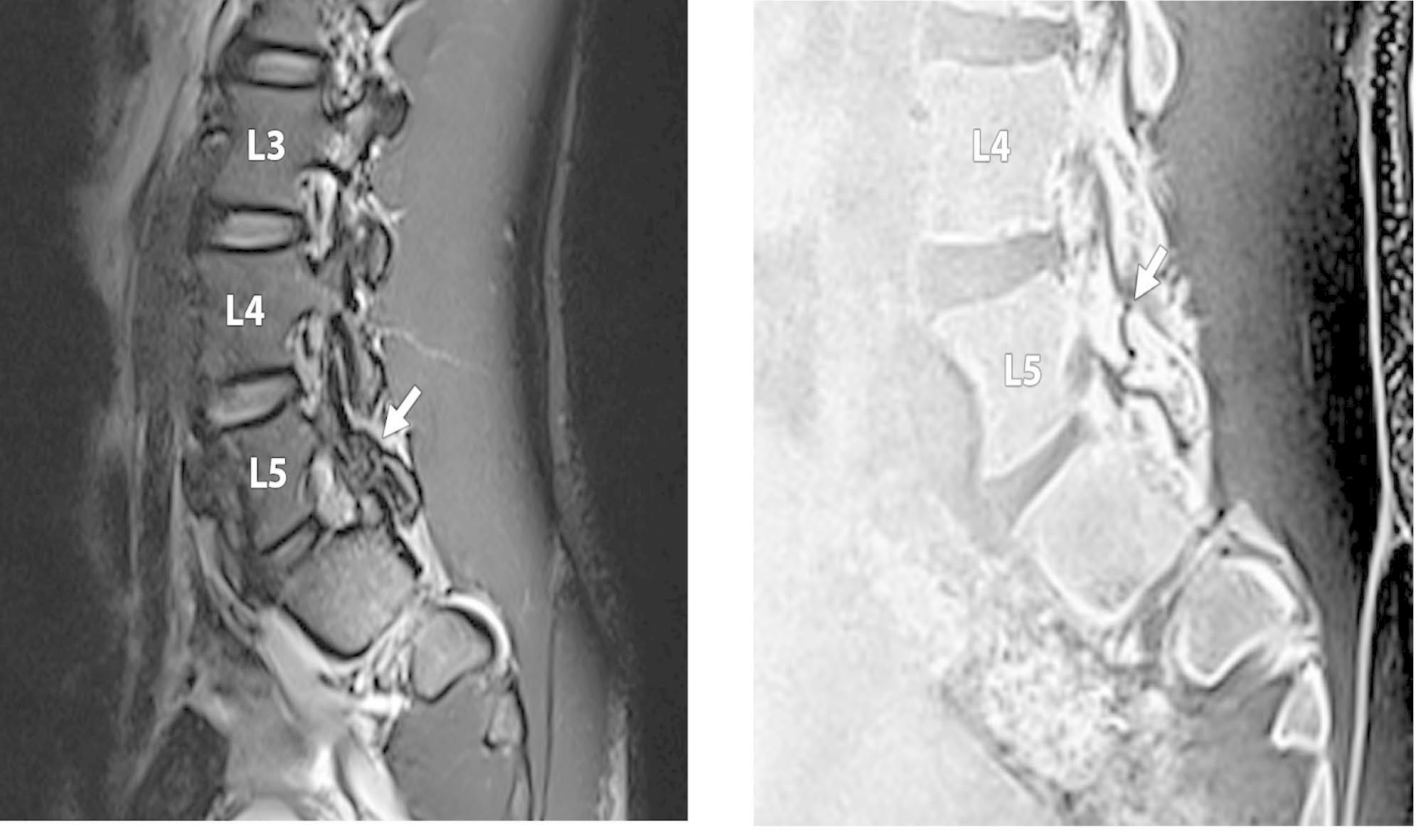
**(a)** 17-year-old male professional footballer with no symptoms. **(a)** Sagittal FST2 sequence demonstrating cortical irregularity of the left L5 pars interarticularis (white arrow), suspicious for a fracture but not definitive. There is no associated bone marrow oedema, suggesting the finding is chronic. **(b)** 17-year-old male professional footballer with no symptoms. **(b)** Sagittal 3D T1 spoiled gradient echo VIBE sequence at a similar level to **(a)**. This sequence definitively confirms the presence of a fracture line (white arrow) within the left L5 pars interarticularis, extending from the inferior to the superior cortex in keeping with a complete fracture. The fracture margins are corticated in keeping with this being a chronic ununited complete pars stress fracture (grade 4) and this is supported by the absence of bone marrow oedema on the FST2 sequence **(a)**

Eighteen players were included in the study. Three of the players (17%) showed no abnormality on MRI; fifteen (83%) showed at least one abnormality, which included facet degeneration, synovial cysts (Fig.1), disc degeneration, disc herniation, and pars injury (Figs.2a-b and 3a-b).

### Facet Joint Arthropathy

One player (6%) showed evidence of mild (Grade 1) facet joint arthropathy. This was at the level of L4/L5 and was left-sided. Three players had bilateral facet joint effusions at one level each, seen at the lower 3 lumbar levels (L3/L4, L4/L5 and L5/S1).

### Synovial cyst formation

Three synovial cysts were identified two players (11%), measuring 3-4mm. Two were left sided (Fig.1) and one was right-sided and all originated from the posterior aspects of the facet joint with no evidence of nerve root compression.

### Pars Interarticularis Abnormality

Four players (22%) were found to have an asymptomatic pars injury. There were five pars interarticulares abnormalities in total, with two abnormalities occurring at the L5 level, and three occurring at the L4 level. The findings were reported in two defenders and two midfielders. Each abnormality was unilateral, with three presenting on the left (left foot dominant n = 2, right foot dominant n = 1) and two on the right (left foot dominant n = 1, right foot dominant n = 1). Two participants required additional, more detailed imaging; one player had an acute incomplete stress fracture of the left L4 pars interarticularis and the other had a chronic ununited pars interarticularis fracture of the left L5 pars interarticularis. A grade 2 acute subtotal stress fracture was seen in three out of the four participants, and a grade 4 chronic stress fracture was seen in the other participant which was confirmed on VIBE sequence as shown in Fig.2b and b.

### Disc degeneration

Seven of the 18 players (39%) demonstrated disc degeneration at one or more levels, involving twelve out of 108 discs (13%) from T12/L1 to L5/S1. The most common level (five out of twelve) for disc degeneration was at L5/S1, with three of these being mild and two moderate. Two out of twelve degenerate discs (17%) were at the L4/L5 level, with one being mild and the other moderate. At L3/L4, there were two mildly degenerate discs. One player had mild degenerate discs at the level of L2/L3 and L1/L2. One participant had mild degeneration at T12/L1 (Fig.2a-b).

### Disc herniation

A total of five disc herniations (28%) were observed in the players, though no high intensity zones were seen in any of the players relating to disc herniation. Three of the herniations were described as circumferential (two at L5/S1, one at L3/L4), and two as central (both at L5/S1 ; Fig.3a-b). No nerve root involvement was seen as a result of disc herniation. None of the discs demonstrated Modic endplate changes. Nine Schmorl’s nodes were seen in four of the five players. One player had multiple limbus vertebrae.

### Other findings

A summary of all of the MRI findings is shown in Table1. One player was found to have a benign haemangioma at the level of S1. Another player had endplate concavities at L2, L3 and L5. Three transitional vertebra were observed, with one lumbarisation, one partial lumbarisation of S1 and one sacralisation of L5. One participant’s images demonstrated bilateral dysplastic pedicles at L5. There was no evidence of any spinal cord abnormalities.

**Table 1:** Distribution of all relevant MRI findings in all participants. The most common findings being disc degeneration and disc herniation

Category		N	Locations	Count	Side	Origin	Grade/Size
Facet Joint Arthropathy		4	T12/L1	0			
			L1/L2	0			
			L2/L3	0			
			L3/L4	2			
			L4/L5	4			
			L5/S1	2			
Synovial Cyst		2	T12/L1	0			
			L1/L2	0			
			L2/L3	0			
			L3/L4	0			
			L4/L5	2	1R,1L	Posterior FJ	3mm, 4mm
			L5/S1	1	1R	Posterior FJ	3mm
Pars Abnormality		4	L1	0			
			L2	0			
			L3	0			
			L4	3	1R, 2L		2,2,2
			L5	2	1R, 1L		4,2
Disc Abnormalities							
	Modic Endplate	0					
	Degeneration	7	T12/L1	1			2*
			L1/L2	1			2*
			L2/L3	1			2*
			L3/L4	2			2*
			L4/L5	2			2,3*
			L5/S1	5			2,2,2,3,3*
	HIZ	0					
	Herniation	5	T12/L1	0			
			L1/L2	0			
			L2/L3	0			
			L3/L4	1	1 Circumferential		
			L4/L5	0			
			L5/S1	4	2 Central, 2 Circumferential		
	Thecal Sac Indentation	0					
Spinal Cord Abnormality							
	Compression	0					
	Myelopathy	0					
	Signal Abnormality	0					
	Syrinx	0					
Other findings							
	Transitional vertebra	3					
	Benign haemangioma	1	S1	1			
	Schmorl’s nodes	4	T11/T12 T12/L1 L1/2 L2/3 L3/4 L4/L5 L5/S1	1 2 1 1 2 1 1			
	Partial lumbarisation	1	S1	1			
	Sacralisation	1	L5	1			
	Endplate concavities	1	L2	1			
			L3	1			
			L5	1			
	Limbus vertebrae	1	T12	1			
			L1	1			
			L4	1			
	Lumbarisation	1	S1	1			
	Bilateral dysplastic pedicles	1	L5	1			

N = number of players with abnormal findings; * = Grading based on Pfirmann et al. (2001)

## Discussion

Asymptomatic lumbar spine pathology was found in 83% of participants within a cohort of elite male academy footballers. The pathologies detected varied with disc degeneration being the most prevalent abnormality seen. Both goalkeepers included in the study had the most extensive disc degeneration (at L4/L5, L5/S1 respectively), and this may be reflective of their specific positional demands (e.g. diving, repetitive lumbar spine extension). Though more research into goalkeepers is needed to confirm this observed trend.

### Clinical implications of findings

These findings highlight the importance of interpreting MRI findings in elite male academy footballers in the context of the clinical signs and symptoms. Team doctors should exercise caution when requesting MRI lumbar spine imaging unless there is a clear clinical indication, and thought has been given as to how to deal with clinically insignificant findings. This study also demonstrates the potential utility of MRI 3D VIBE sequencing to diagnose and assess healing of pars injuries in elite footballers and could negate the need to expose players to ionising radiation (e.g., CT), however more robust studies would be needed to confirm this finding.

### Are these findings normal in young athletes?

These findings are consistent with an emerging body of research which has identified that both symptomatic and asymptomatic lumbar spine pathology is common amongst young athletes and the active population [18]. Disc degeneration amongst this cohort of professional footballers was the most prevalent abnormality seen (39%), and was marginally higher than that seen in non-athletes of a similar age profile (31–37%) [19, 20]. Other youth sport athletes have shown higher rates of disc degeneration, e.g. tennis players (62%) and elite gymnasts (75%) [4, 20]. The prevalence of facet joint arthropathy (6%) was considerably lower than that seen amongst tennis players (90%), but mirrored findings in the general population for those aged under 30 years of age (4–9%) [4, 19]. No data from the age-matched general population is, to the authors’ knowledge, available for comparison. The prevalence of asymptomatic pars injury, although reasonably high (22%), was less than that seen in diving (35%) or cricket (32%) where repetitive spinal flexion, rotation and hyperextension actions increase the risk of pars injury [21, 28] It therefore seems that partaking in high volumes of highly specialized sport from a young age increases the risk of both asymptomatic and symptomatic lumbar spine pathologies. The risk of these pathologies appears to be dependent on the sport and its sport-specific demands on the lumbar spine (e.g., repeated spinal rotation, lumbar flexion or rapid sprint decelerations).

### Should we be concerned about the findings?

Four asymptomatic participants were identified to have pars fractures on MRI. There was no clear trend between the player’s dominant foot and the side on which the pars fracture presented, and their playing position did not appear to be contributory. Detailed clinical examination may have elicited positive findings in these cases, however, the absence of symptoms may also be explained by different pain coping mechanisms among elite athletes, the possibility that the players may be truly asymptomatic or that the imaging changes precede the symptoms as has been shown in cricket [9, 21, 22] One player had endplate concavities at L2, L3 and L5 which can be suggestive of sickle cell disease [23]. In accordance with the pre-established protocol for managing incidental findings, a sickle screen blood test was performed which was negative.

It is unknown whether these findings are unique to the team assessed or a common trend in football. Until larger studies identify prevalence values, these findings of concern highlight the importance of regularly screening youth academy footballers despite being asymptomatic, similar to standards already in place in sports such as cricket [9]. Considering the burden of these injuries in Academy footballers, such screening may prove beneficial to the long-term health and performance of players [2].

### Key recommendations

As asymptomatic lumbar spine pathologies have been shown to be common when partaking in high volumes of a highly specialised sport from a young age, it is important to not just screen but also aim to minimise the risk of these pathologies. The football physician should ensure early identification of symptoms to enable appropriate strength training and load management in order to prevent progression to a significant time-loss injury. [24]. With specific reference to preventing pars stress fractures, other plausible interventions may include Vitamin D supplementation and ensuring adequate energy availability to meet training demands [25, 26]. This is particularly important as, prolonged absence through injury leads to significant ‘lost development time’ for academy players which can hinder career progression [27].

### Strengths and limitations

This is the first study to describe the MRI lumbar spine findings of asymptomatic elite male adolescent footballers. A rigorous study design protocol was adhered to using the STROBE checklist. Novel and emerging imaging techniques (e.g., MRI 3D VIBE) were employed to describe common injuries (e.g., pars).

The most obvious limitation to this study is the small sample size; although this is comparable to other studies investigating elite sporting populations [4, 5, 7]. Our study may have benefitted from including more extensive baseline data such as height, weight, peak height velocity, ethnicity and Vitamin D levels but with the low sample size any investigated associations would have been exploratory and limited. Future studies should look to include a larger sample size, from a variety of different clubs to more accurately identify prevalence and to explore the relationship between clinical examination findings and imaging results. Long-term follow-up of these athletes over the course of their career may also provide insight into the specific effects of professional football on spine health, as well as helping to predict which imaging findings may precede symptom-onset.

## Conclusion

Asymptomatic lumbar spine pathology is a common (83%) MRI finding among elite adolescent footballers, though research has shown that partaking in high volumes of highly specialised sport from a young age increases the risk of both asymptomatic and symptomatic lumbar spine pathologies. A range of unsuspected findings on MRI of the lumbar spine was therefore common in elite adolescent footballers. For medical staff, it is important not only to screen but to also to aim to minimise the risk of these pathologies and thereby decrease the trend of making asymptomatic lumbar spine pathologies in young athletes ‘the new normal’.

## Data Availability

The datasets generated and analysed during the current study are not publicly available due to the potential to compromise individual privacy but are available from the corresponding author on reasonable request.
